# Keep Calm and Carry On: Improved Frustration Tolerance and Processing Speed by Transcranial Direct Current Stimulation (tDCS)

**DOI:** 10.1371/journal.pone.0122578

**Published:** 2015-04-02

**Authors:** Christian Plewnia, Philipp A. Schroeder, Roland Kunze, Florian Faehling, Larissa Wolkenstein

**Affiliations:** 1 Department of Psychiatry and Psychotherapy, Neurophysiology & Interventional Neuropsychiatry, University of Tübingen, Calwerstrasse 14, 72076 Tübingen, Germany; 2 Werner Reichardt Centre for Integrative Neuroscience, University of Tübingen, Otfried-Müller-Str. 25, 72076 Tübingen, Germany; 3 Department of Psychology, Clinical Psychology and Psychotherapy, University of Tübingen, 72076 Tübingen, Germany; University of British Columbia, CANADA

## Abstract

Cognitive control (CC) of attention is a major prerequisite for effective information processing. Emotional distractors can bias and impair goal-directed deployment of attentional resources. Frustration-induced negative affect and cognition can act as internal distractors with negative impact on task performance. Consolidation of CC may thus support task-oriented behavior under challenging conditions. Recently, transcranial direct current stimulation (tDCS) has been put forward as an effective tool to modulate CC. Particularly, anodal, activity enhancing tDCS to the left dorsolateral prefrontal cortex (dlPFC) can increase insufficient CC in depression as indicated by a reduction of attentional biases induced by emotionally salient stimuli. With this study, we provide first evidence that, compared to sham stimulation, tDCS to the left dlPFC enhances processing speed measured by an adaptive version of the Paced Auditory Serial Addition Task (PASAT) that is typically thwarted by frustration. Notably, despite an even larger amount of error-related negative feedback, the task-induced upset was suppressed in the group receiving anodal tDCS. Moreover, inhibition of task-related negative affect was correlated with performance gains, suggesting a close link between enhanced processing speed and consolidation of CC by tDCS. Together, these data provide first evidence that activity enhancing anodal tDCS to the left dlPFC can support focused cognitive processing particularly when challenged by frustration-induced negative affect.

## Introduction

Effective, goal-directed information processing and behavior is constantly challenged by distraction. Although quick responses to potentially threatening or otherwise salient stimuli are essential for survival and thus adaptive in a dynamic environment, these momentary (re-)actions often interfere with ongoing cognitive processes [1–3]. Therefore, task-oriented behavior requires cognitive control (CC) to regulate attention deployment. However, dysfunctional CC is regarded as a critical pathophysiological mechanism in various psychiatric disorders and thus poses a serious health issue [4,5]. Particularly depressive disorders are characterized by increased elaboration of negative information and by difficulties to disengage from negative content [6,7]. Therefore, current clinical research increasingly includes this aspect in the optimization of existing and the development of new treatment approaches [8–10].

A large body of evidence indicates that ‘top-down’ CC critically relies on the activity of frontopariental regions [11–13]. The presence of emotionally distracting stimuli during a challenging working memory task for instance was associated with reduced performance, deficient activity in the dorsolateral prefrontal cortex (dlPFC) and an increase in amygdala activity [14,15]. Consistently, it has been suggested that dlPFC activity contributes to the preservation of cognitive task performance when emotionally distractive stimuli occur [16].

Currently, non-invasive brain stimulation methods are increasingly used to investigate the physiology and malleability of this neurocognitive network [17]. For this purpose, particularly transcranial direct current stimulation (tDCS) is applied, a technique to transiently modulate cortical activity by means of a resting-membrane shift induced by a weak, 1–2 mA current [18,19]. Reliable, polarity-specific, activity-dependent and behaviorally relevant effects of this intervention are extensively described [20–23]. Anodal, activity enhancing tDCS, was recently shown to ameliorate dysfunctional cognitive control on the processing of distractive emotional pictures in patients with major depression [24]. Moreover and consistent with the notion of a relevant role of the dlPFC for CC, it was also demonstrated that a depression-like negativity bias can be induced in healthy subjects by reducing dlPFC activity with cathodal tDCS [25]. However, crucial challenges for CC can also arise from stress, negative cognitions, self-referential thinking and rumination [5,26], likewise associated with reduced dlPFC activity [27,28] and impaired executive functioning [29]. In addition, increasing cognitive load can also negatively influence inhibitory control on emotional distraction [30,31].

With this study, we set out to assess the malleability of CC on mutual interactions between task-induced negative affect and executive functions by brain stimulation. Anodal tDCS was used to enhance activity of the left dlPFC and to test its effect on CC as quantified by means of processing speed in the presence of frustration and negative affect [10,32]. We hypothesized that the enhancement of dlPFC activity would improve CC and that the consequential suppression of task-induced negative feelings and associated cognitions would result in improved task-performance.

## Methods and Materials

### Participants

Twenty-eight male and healthy participants (mean age = 27.9 years, SD = 9.3) took part in one experimental session of 60 minutes duration for monetary compensation. All participants were right-handed according to the Edinburgh Handedness Inventory (LI > 75) [33]. The study was approved by the local ethics committee and was conducted in compliance with the Declaration of Helsinki. None of the participants had a history of mental or neurological illness; further exclusion criteria were: epilepsy, pacemaker, metal implants, use of CNS-acting drugs or recreational substances, and cognitive impairments.

### Adaptive Paced Auditory Serial Addition Task (PASAT)

The computer-based adaptive Paced Auditory Serial Addition Task (PASAT) has been originally designed to test patients with head trauma [34] and was later modified as a tool to elicit stress under standardized conditions while measuring processing speed and adaptation capacity [35]. A clear involvement of the left dlPFC in the performance of the PASAT has already been demonstrated [36]. In the task used in this study, single digits were presented via headphones. The participants were asked to add each new digit to the preceding digit and indicate the correct result by a click on the corresponding number on a computer screen. Initially, the inter-stimulus- interval (ISI) between digit presentations was set at 3 seconds and adapted according to task performance: After four consecutive correct answers, the ISI decreased by 0.1 second, and after four consecutive incorrect answers, the ISI increased by 0.1 second. Therefore, the speed of stimulus presentation and therefore task difficulty was escalated to the individual maximum. Visual feedback was given on correct, incorrect and missed answers.

### tDCS

**Fig 1:**
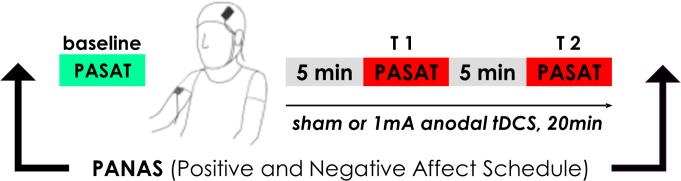
Experimental procedure. All participants completed the ‘Positive and Negative Affect Schedule’ (PANAS) and then performed a baseline measurement of the adaptive Paced Auditory Serial Addition Task (PASAT) without tDCS. Then either sham or anodal tDCS was initiated. For the first 5 minutes of stimulation, participants were instructed to relax. Next, a second and third measurement of PASAT performance were conducted for 5 minutes each, with a break of 5 minutes in between. Afterwards, stimulation was terminated and participants completed the PANAS post questionnaire.

Transcranial direct current was delivered continuously by a CE-certified stimulator (DC-STIMULATOR MC, NeuroConn GmbH, Ilmenau, Germany) using a pair of saline-soaked 5x7cm sponge electrodes. To increase activity of the left dlPFC, the anode was placed on the scalp over F3 according to the international 10–20 system of electrode placement [37] and fixated with two rubber bands. The reference electrode (cathode) was placed on the contralateral deltoid muscle to avoid unwanted opposite polarization of another brain area [24,38]. Stimulation was started after participants completed the baseline PASAT block. The following test PASAT blocks (T1, T2) were completed during (active or sham) stimulation (Fig 1). Active tDCS was administered for 20 minutes with a constant current of 1 mA and a linear fade-in/fade-out phase of 5 seconds. For sham stimulation, the same electrode placement was used but the current was only applied for 30 seconds at the onset of the sham session and then ramped down, thereby eliciting a transient tingling experience comparable to that elicited by verum stimulation without any effects in the brain [39].

### Positive and Negative Affect Schedule (PANAS)

The PANAS is a self-report measure of affect consisting of twenty single adjectives that describe affective states [40]. It is divided into two subscales of 10 items each, measuring positive and negative affect. Items are rated on a 5 point Likert scale ranging from 1 ‘not at all’ to 5 ‘very much’. Its high construct validity was shown in a large sample [41]. We used the German version as translated by Krohne, Egloff, Kohlmann, & Tausch [42]. Both, the used German words and their re-translations following Krohne and colleagues are reported throughout the results section. Participants indicated to what extent they experienced each of the twenty affective states at this precise moment.

### Procedure

The study was designed as a single-session sham-controlled experiment. All participants underwent the same experimental protocol, but only half of them (n = 14) received anodal tDCS whereas the other half (n = 14) received sham stimulation. The experimental procedure is illustrated in Fig 1. Prior to performing the baseline and two test blocks of the PASAT, participants were equipped with tDCS electrodes, seated comfortably at a distance of ~60cm to a 17” monitor and closed headphones were put on, preset at a clear and constant sound volume. The ‘Positive and Negative Affect Schedule’ (PANAS) was administered immediately before the baseline PASAT block and immediately after the second test PASAT block.

### Statistical Analyses

Mean inter-stimulus-intervals were extracted from the adaptive PASAT task and were subjected to a 2x2 ANCOVA comprising the within-subjects factor block (block 1 vs. block 2) and the between-subjects factor stimulation (sham vs. anodal). Baseline PASAT performance prior to the beginning of the stimulation was tested for group differences first and consecutively included as covariate. For data from the PANAS questionnaire, we subjected the each 10 individual scores of positive and negative affect to two separate mixed MANOVAs comprising the factors time (pre vs. post stimulation) and stimulation as referred above. Significant effects (Wilk’s lambda criterion) were followed up by separate 2x2 ANOVAs, Bonferroni-corrected for multiple comparisons and FWE inflation. Next, we correlated mean PASAT improvement with differential affect changes as obtained from the PANAS analysis using Spearman’s Rho as nonparametric rank correlation coefficient for Likert-scale differences. PASAT improvement was defined as: ΔISI = Mean(ISI_Block1_; ISI_Block2_)−ISI_Baseline_. Finally, and as a control measure, we subjected absolute error and omission frequencies to the ANCOVA and analyzed the corresponding affect correlation as described above.

## Results

### Study sample

Participants were randomly assigned to the active or sham group. There was no significant difference between groups in age (tDCS: 28.9 years, SD = 10.6; sham: 26.9 years, SD = 8.0; *t*(26) = 0.58, *p* = .56) and education (predominantly university students).

### Processing speed (PASAT)

**Fig 2:**
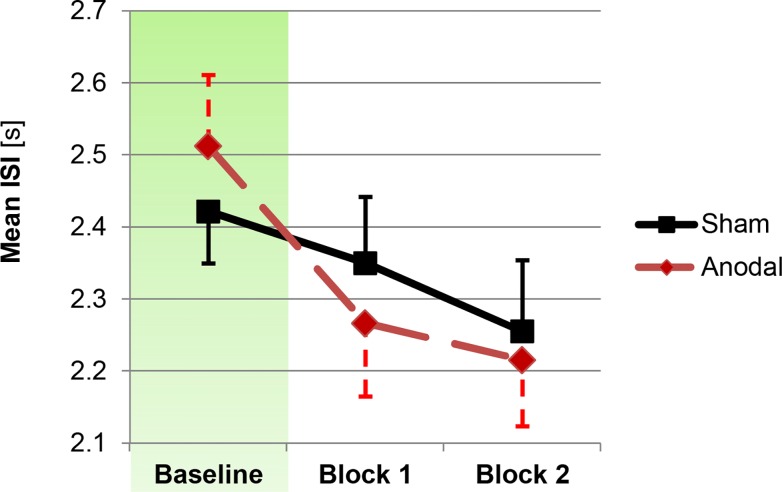
Changes in processing speed as a function of stimulation. Anodal stimulation led to a significant stimulation main effect in the corresponding block x stimulation ANCOVA, driven by shorter ISIs in the stimulation group. Error bars reflect standard errors of the mean.

Mean ISIs from the PASAT task blocks and for sham and anodal stimulation groups are depicted in Fig 2. In the baseline block, no significant group difference was found, *t*(26) = 0.71, *p* = .48. For the 2x2 ANCOVA model addressing differential effects during the two stimulation blocks, the baseline covariate was highly significant, *F*(1,25) = 100.95, *p*<.001, η_p_² = 0.80. There was no main effect of block, *F*(1,25)<1, *p* = .90. Importantly, a significant main effect of stimulation emerged, *F*(1,25) = 5.55, *p* = .027, η_p_² = 0.18, driven by shorter ISIs in the anodal stimulation group (2241 *ms*, SE = 98 *ms*) than in the sham stimulation group (2303 *ms*, SE = 97 *ms*) during the two test blocks. None of the interaction effects yielded significance, *p*s>.42.

### Affect changes / PANAS

#### Positive Affect

Overall, positive affect did not change from pre- to posttest and the main effect of time was not significant, *F*(1,26)<1, *p* = .35. There was no effect of stimulation, *p* = .66, and no significant interaction, *p*s>.13.

#### Negative Affect

For the negative affect scale, the main effect of time approached significance, *F*(1,26) = 3.62, *p* = .068, η_p_² = 0.12. There was a group-specific and item-specific modulation of negative affect over time, expressed in a significant three-way interaction term, *F*(9,18) = 3.18, *p* = .018, η_p_² = 0.61. We followed up on this interaction by conducting separate 2x2 ANOVAs (time x stimulation) for each item. The item-specific results of the negative affect scale were Bonferroni corrected for α-error inflation $\text{(}p_{\text{crit}}=\frac{\alpha }{\text{10}}=0.005\text{)}$ and are reported exhaustively in Table 1.

**Table 1:** Negative affect changes associated with stimulation condition.

**Item (German)**	**Stimulation**				**Interaction (time x stimulation)**	
	**sham**		**anodal**		***F***	***p***
	**pre**	**post**	**pre**	**post**		
distressed (bekümmert)	1.43	1.50	1.50	1.14	*2*.*07*	.*16*
upset (verärgert)	**1.36**	**2.79**	**1.29**	**1.50**	***12*.*32***	.002*
guilty (schuldig)	1.14	1.07	1.00	1.43	*4*.*39*	.*05*
scared (erschrocken)	1.00	1.21	1.07	1.29	*0*	*1*
hostile (feindselig)	1.43	1.64	1.29	1.71	*0*.*38*	.*54*
irritable (gereizt)	2.00	2.57	1.86	1.57	*3*.*49*	.*07*
ashamed (beschämt)	1.36	1.57	1.14	1.21	*0*.*33*	.*57*
nervous (nervös)	1.93	1.64	2.14	1.64	*0*.*45*	.*51*
jittery (durcheinander)	1.43	1.29	1.43	1.43	*0*.*48*	.*49*
afraid (ängstlich)	2.36	2.50	1.86	2.14	*0*.*10*	.*75*

Mean ratings are reported for each item; results refer to the individual 2x2 ANOVAs comprising the factors time (pre vs. post task) and stimulation (anodal vs. sham) for each of the 10 negative affect items of the PANAS questionnaire. Interactions are considered significant and marked with asterisks where *p*<.005 (Bonferroni-corrected for multiple testing).

**Fig 3:**
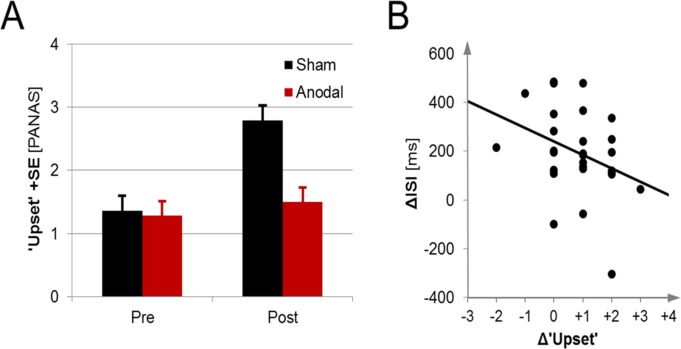
Feeling ‘upset’ and processing speed. Whereas participants in the sham stimulation group felt more upset after the experiment, this increase was alleviated in the anodal stimulation group (Panel A). Increase in feeling upset was correlated with less processing speed improvements as measured by the PASAT inter-stimulus interval (ISI; Panel B). Error bars represent standard errors of the mean.

Non-significant trends emerged for the items ‘guilty (‘schuldig’), *F*(1,26) = 4.39, *p* = .046, η_p_² = 0.15, and ‘irritable’ (‘gereizt’), *F*(1,26) = 3.49, *p* = .073, η_p_² = 0.12. Most importantly, a significant interaction indicating differential affect changes emerged for the item ‘upset’ (‘verärgert’), *F*(1,26) = 12.32, *p* = .002, η_p_² = 0.32 (Fig 3A). Follow up paired t-tests detected a significant increase in upset for the sham stimulation group, *t*(13) = 5.70, *p*<.001, *d* = 1.48, but not in the anodal stimulation group, *t*(13)<1, *p* = .39.

### Negative affect and processing speed

Finally, to address the assumed relationship between negative affect and reduced processing speed, we correlated changes in feeling upset with ISI changes from baseline to the test blocks under stimulation. In line with our hypothesis, a significant negative rank correlation coefficient emerged, r = -.38, *p* = .022, signaling higher ISIs (or slower processing) with increased upset (Fig 3B).

### Error and omission frequencies

Aggregated over both test blocks (T1, T2), in the sham condition 88.93 (SE = 2.34) errors (incl. omissions) were made as compared to 95.86 (SE = 2.40) errors in the anodal group. Participants error frequencies did not differ in the preceding baseline block, *t*(26) = .06, *p* = .95. In the corresponding ANCOVA, a marginal significant main effect of stimulation, *F*(1,25) = 4.14, *p* = .053, η_p_² = 0.14 was found. Neither the main effect of block nor the two-way interaction approached significance, all *p*s>.54.

## Discussion

With this study, we provide first evidence that the enhancement of activity in the left prefrontal cortex by anodal tDCS during an adaptively challenging attention task improves performance parallel to, and in correlation with the suppression of specific task-induced negative affect. These data can be interpreted as a tDCS-supported shift of processing resources towards task-oriented performance away from preoccupation with task-related negative affect and cognition. Thereby they extend the notion of enhanced CC by prefrontal activation [24] to internally generated distress-related distractors. More specifically, by demonstrating that tDCS-induced higher performance is associated with a lesser degree of feeling ‘upset’ with the task, these data may exemplify a subjective, experiential aspect of enhanced CC in challenging operations. Not least, these findings substantially support the concept of a tDCS-enhanced CC training as a new pathophysiology-based treatment approach of disorders associated with dysfunctional CC [43–45].

Comprehensive previous work has highlighted the relevance of prefrontal activity for CC which is critical to select the goal-relevant features of experience [1,12]. It has therefore been proposed that a neuronal system including frontoparietal cortical regions allows for a flexible regulation of attention deployment by biasing processes in other brain systems towards internal intentions [11]. Anyway, more or less salient stimuli from the environment or from internally generated drives continuously strive for attention [3]. Since processing in the brain is highly competitive with different pathways mediating different aspects of information, the winners are those with the strongest sources of support [11]. Accordingly, increased activation of the dlPFC by anodal tDCS might strengthen its function to avert attention from affective reactions induced by performance errors and thus maintain goal-directed processing. The most plausible mechanism, we assume, includes a more effective inhibition of the emotional appraisal systems including the amygdala and ventromedial prefrontal cortex by an enhanced or more effective activation of the dlPFC [15,16,46]. The malleability of this system by training and tDCS and its combination has been demonstrated by previous studies [24,25,47–49]. Our results support and extend previous findings demonstrating enhanced working memory performance under anodal tDCS [50,51] as well as improved CC on distraction by emotionally salient pictures in patients with major depression [24].

Alternatively, an enhancement of working memory performance by anodal tDCS to the dlPFC might merely be paralleled or followed by less negative emotional reactions. In this regard, it is important to consider that in the adaptive form of the PASAT [10], higher processing speed (i.e. performance) is associated with a larger number of tasks (and errors) and consequently a comparable if not even larger amount of negative feedback. Therefore both groups, similar to previous studies [32,52], were needled to become ‘upset’ with and by the annoying task through the belief or the feeling not to perform well enough. Actually, while sham-treated participants reported this upsetting experience, participants ‘kept calm’ in the anodal tDCS condition. Particularly this flexible cognitive-affective appraisal of actual task performance was linked with dlPFC function before [46,53,54] and may thus represent the experiential aspect of flexible CC, critically participating in the regulation of sustained attention.

It also has to be considered that anodal tDCS may have generally reduced negative affect with a secondary effect on CC. But, our data do not support this notion. Although ‘distress’ was inhibited by anodal tDCS, a general suppression of negative affect by tDCS as quantified by the PASAT negative affect subscore was not observed. However, the complex interaction between affect, attention and cognitive processing has already been comprehensively described [55–58] and impedes inferences about the primary target of tDCS. Patients with depression show clear deficits in executive function, memory and attention relative to controls [59,60]. Negative emotional states in general have been associated with decreased cognitive performance [56] and with reduced activity in the left dlPFC [61–64]. Eventually, the aversive reactions to frustration as induced in our study could trigger adaptive changes in behavior since subjects will work to avoid this frustration by increased effort and attention. However, if adaption fails, resulting failure-related thoughts and associated worries can pose a secondary workload factor drawing resources away from the primary task and devoting them to self-focused negative cognitions [65–67]. In that regard, the PASAT has been described as an effective tool to measure cognitive performance, particularly under distraction by negative emotional states associated with frustration [10,32,52,68]. Therefore, this model appears to be suitable to measure the effects of tDCS on CC of internal negative feedback and associated distraction to maintain focused attention [67]. Healthy populations as investigated in this study disclose decrements in attentional processing by internal negative feedback most likely to a less severe degree than clinical populations of anxious or depressed patients [49]. Therefore, the present observations point towards a promising therapeutic use in these disorders via an amelioration of insufficient CC on self-reflective rumination [5,69]. Still, recent attempts to use the combination of PASAT and tDCS as a tool to treat insufficient CC in depressive subjects have provided inconsistent results particularly regarding the enhancing effects of tDCS on training of CC over multiple session [43,45,70]. To establish a complimentary pathophysiology-based antidepressant treatment, further insights in the mechanisms and interactions of non-invasive brain stimulation (NIBS) and specific training tasks are continuously gathered. Regarding methodological limitations of our approach, it is important to consider the low topographic specificity due to the size of the stimulation electrode (35 cm^2^) and the tDCS-associated network effects that do not allow for an exact localization of the modulation effect [17]. However, to maximize the spatial resolution, an extracephalic cathodal reference electrode was used to preclude a simultaneous opposite polarization of another brain area [22,24,25]. The sham-control condition was realized by fade-in, short stimulation, and fade-out which has been shown to be reliable for naïve and experienced subjects and is a frequently used, well-established control condition [71].

To account for the individual variance in PASAT processing speed and to focus on the tDCS effects, we partialled out baseline PASAT performance in our statistical analysis. This approach did not reveal a significant interaction between the two test PASAT blocks (1, 2) and stimulation, but it appears that the active and the sham group reached nearly the same performance level in block 2. Therefore, it is definitely possible that the effect is predominantly due to changes from baseline to the first PASAT block and dependent on the initial task performance level. Further studies will be needed to investigate the interaction between tDCS and task proficiency or training.

In sum, our data provide proof of concept for an improved CC over task-induced negative emotions by anodal electric stimulation of the left dlPFC. While extant research on modulation of CC by brain stimulation has focused primarily on clinical populations, our data extend the knowledge on plasticity and malleability of healthy CC on internal emotional distraction. In the clinical domain, these findings can support ongoing research on the specific combination of brain stimulation and cognitive training [43,45,70]. On the one hand synergistic effects of dlPFC function reactivation in depressive disorders might significantly support treatment [47]. On the other hand, CC training supported by brain stimulation might facilitate and consolidate the experience of emotion control [9]. Of particular importance for a more detailed understanding of mechanisms and clinical utility future studies will explore predictors and mediators of brain stimulation effects. In this regard, imaging [72,73], genetic [74] and neurophysiological data [75] will deepen the knowledge on the physiological mechanisms of CC to individualize and improve the effects of brain stimulation as a new tool to ameliorate deficient CC in patients with psychiatric disorders.

## Supporting Information

S1 Dataset Data underlying the findings. (XLS)
